# Microwave-Assisted Grafting of Coal onto Nitrogen-Doped Carbon Dots with a High Quantum Yield and Enhanced Photoluminescence Properties

**DOI:** 10.3390/molecules29061349

**Published:** 2024-03-18

**Authors:** Chong Shi, Xian-Yong Wei

**Affiliations:** 1 Jiangsu Province Engineering Research Center of Fine Utilization of Carbon Resources and Key Laboratory of Coal Processing and Efficient Utilization, Ministry of Education, China University of Mining & Technology, Xuzhou 221116, China; shichong0901@163.com; 2 Key Laboratory of Chemistry and Chemical Engineering on Heavy-Carbon Resources, Yili Normal University, Yining 835000, China; 3 State Key Laboratory of Chemistry and Utilization of Carbon-Based Energy Resources Jointly Built by Xinjiang Uyghur Autonomous Region and Ministry of Science and Technology, Key Laboratory of Coal Clean Conversion & Chemical Engineering Process (Xinjiang Uyghur Autonomous Region), College of Chemical Engineering, Xinjiang University, Urumqi 830046, China

**Keywords:** microwave, anthracite, lignite, nitrogen-doped carbon dots, solvothermal approach, quantum yield, yield

## Abstract

The fluorescent nitrogen-doped carbon dots (N-CDs) were synthesized via a facile one-pot solvothermal process using coal (Jin 15 Anthracite and Shaerhu lignite) as raw materials and dimethyl formamide (DMF) as the solvent, employing a microwave pyrolysis method. This approach demonstrates remarkable efficacy in the development of nitrogen-doped carbon dots (N-CDs) with a high quantum yield (QY). The N-CDs prepared have strong photoluminescence properties. Moreover, the obtained N-CDs emit blue PL and are easily dispersed in polymethyl methacrylate (PMMA), preserving the inherent advantages of N-CDs and the PMMA matrix. The JN-CDs exhibit a high quantum yield (QY) of 49.5% and a production yield of 25.7%, respectively. In contrast, the SN-CDs demonstrate a quantum yield of 40% and a production yield of 35.1%. It is worth noting that the production yield and quantum yield of coal-based carbon dots are inversely related indices. The lower metamorphic degree of subbituminous coal favors an enhanced product yield, while the higher metamorphic degree of anthracite promotes an improved quantum yield in the product, which may be attributed to the presence of amorphous carbon within it. Consequently, we propose and discuss potential mechanisms underlying N-CD formation.

## 1. Introduction

Carbon dots (CDs) are a rising star in the family of carbon nanoparticles and have garnered extensive attention due to their luminescent properties [1,2,3,4,5,6,7,8]. The past decade has witnessed rapid development in carbon dots (CDs) owing to their diverse sources, including (a) fullerene; (b) graphite; (c) graphene oxide; (d) carbon nanotubes; (e) carbohydrates; etc., as well as significant advantages, such as (a) elemental abundance; (b) hypotoxicity; (c) chemical inertness; (d) outstanding biocompatibility; (e) unique physical properties; (f) affordability; and so on [9,10,11,12,13,14,15,16]. Various methods are commonly used to synthesize fluorescent CDs, such as (a) electrochemical synthesis processes; (b) chemical oxidation methods; (c) hydrothermal cutting strategies; (d) carbonizing organics routes; (e) laser ablation of graphite; (f) ultrasonic methods; (g) hydrothermal; (h) solvothermal; (i) microwave-assisted pyrolysis; etc. [17,18,19,20]. Nevertheless, most of these developed techniques may be unsatisfactory owing to (a) the complexity of the procedures; (b) expensive carbon source; (c) difficult post-processing, which limit the development and applications of CDs [15]. In particular, the fluorescence quantum yield of most fluorescent carbon quantum dots is usually less than 50%, which is relatively low compared to conventional semiconductor quantum dots. Particularly noteworthy among the available carbon sources is coal for its distinctive and appealing superiority [21,22,23,24,25,26,27,28,29,30].

The energy source coal, for its structural feature, is mostly used for combustion. The microscopic examination reveals that coal is composed of polymerization aromatics nanoscale carbon crystal domains and aliphatic amorphous carbon. Within coal, there are abundant nanoscale carbon crystal areas, with each area being the size of a quantum dot. The shape and size of these nano-crystallites in coal are determined by the coalification process and their maturity level. If these domains can be efficiently exfoliated to form carbon quantum dots, coal could serve as an ideal source of carbon for their preparation [2].

For instance, Ye et al. employed a wet chemical method to synthesize graphene quantum dots via the acidic oxidation heat treatment of coal [28]. In a previous study by Li et al. [1], ethylenediamine-grafted carbon dots were synthesized through acid oxidation of coal followed by surface grafting, resulting in a high quantum yield (QY) [8]. Coal-based fluorescent carbon dots with controllable sizes were prepared by combining carbonization and acid oxidation etching [31]. However, all the aforementioned methods involve the use of concentrated acids, which not only makes them time-consuming and environmentally unfriendly but also leads to a relatively low QY. On one hand, subdomains formed during acid oxidation treatment are detrimental to energy efficiency and result in weak intrinsic emission [32]. On the other hand, acidic oxidation introduces various oxygen-containing functional groups on carbon dots that can cause non-radiative recombination of electron–hole pairs and, consequently, lead to a relatively low QY [33,34].

Microwave-assisted synthesis of CDs is an efficient methodology. This significant finding garnered widespread attention in the field of microwave-accelerated organic reactions. Numerous experimental studies have substantiated that employing microwave technology for organic reactions yields reaction velocities tens or even thousands of times higher compared to traditional heating methods [9]. In comparison to conventional synthetic approaches, the microwave-mediated method offers several advantages, including (a) accelerated heating rate; (b) enhanced heat energy utilization efficiency; (c) heightened reaction sensitivity; (d) production of high-quality products; and (e) improved safety and health considerations. Furthermore, microwave irradiation typically yields CDs with a high quantum yield [7]. Consequently, microwave-assisted organic reactions have witnessed rapid advancements.

The coal structure consists of sp2 carbon domains and interconnected layers through bridging bonds. A one-pot solvothermal approach can be employed to disperse the light components in the solvent, while simultaneously utilizing the stripping effect of solvothermal treatment to cleave oxygen bridge bonds and facilitate the synthesis of carbonaceous nanoparticles derived from coal [14]. In this study, fluorescent nitrogen-doped carbon dots (N-CDs) were synthesized from coal (Jin 15 Anthracite and Shaerhu lignite) as raw materials and dimethyl formamide (DMF) as a solvent using a microwave pyrolysis process. The resulting N-CDs exhibited strong photoluminescence properties [35,36]. Specifically, the JN-CDs demonstrated a high quantum yield (QY) of 49.5% with a production yield of 25.7%. Similarly, the SN-CDs displayed a quantum yield of 40% and a production yield of 35.1%. Both JN-CDs and SN-CDs were obtained through rotary evaporation, allowing for the recycling of the solvent used in their synthesis. This effective, environmentally friendly, and sustainable approach offers great potential for producing high-quality fluorescent CDs [37,38,39]. It is anticipated that this unique strategy for fabricating high-quality fluorescent CDs will open up new possibilities for their practical application.

## 2. Results

The yield of the CDs was determined by two methods: fluorescence quantum yield (QY) and production yield. For production yield, N-CD suspension was obtained using rotary evaporation. The fluorescence quantum yield (QY) of the CDs was also measured as a means to assess their yield. In terms of N-CDs, their observed fluorescence QY was measured under 340 nm excitation (refer to Table S2). Both SN-CDs and JN-CDs exhibited enhanced photoluminescence with a high QY of 49.5% and a production yield of 25.7% for JN-CDs, as well as a high QY of 40% and a production yield of 35.1% for SN-CDs, surpassing those achieved by DMF-derived carbogenic dots (with a QY of 18.6%) and coal-based CDs (0.81%) [2] (Table 1, Tables S1 and S2). The carbonaceous core of coal-based carbon dots, prepared via the solvothermal method, is a microregion composed of a few layers of graphene fragments and limited by carbon. These π electrons form relatively complete conjugated structures, resulting in intrinsic fluorescence. Simultaneously, the surface contains nitrogen-containing functional groups that resemble surface and edge defects observed in organic fluorophores formed by small molecules. The carbon dots derived from DMF exhibit similarities to those obtained through bottom-up methods using other small molecules as reported in the literature, with their luminescent function attributed to specific organic luminescent groups. In addition to the relatively complete carbonaceous core achieved through etching, coal-based carbon dots prepared via acid oxidation possess abundant oxygen-containing functional groups on their surfaces, which can be passivated through reduction using reducing agents or grafting electron donor groups to obtain fluorescent carbon dots with high luminescence quantum yield [1].

Both types of coal samples underwent identical conditions initially, aiming to enhance the dimensions and chemical characteristics of graphitic crystallites.

Subsequent analysis utilizing scanning electron microscopy (SEM) unveiled a noteworthy decrease in particle size down to several micrometers (Figure S1), accompanied by the presence of flake-shaped morphologies. The original anthracite exhibits a non-uniform shape and size distribution, yet it possesses a relatively homogeneous structure with micron-sized particles. This significant increase in the samples’ specific surface area produced structural defects in the coal, resulting in a higher activity of selective grafting with DMF. 

**Figure 1:**
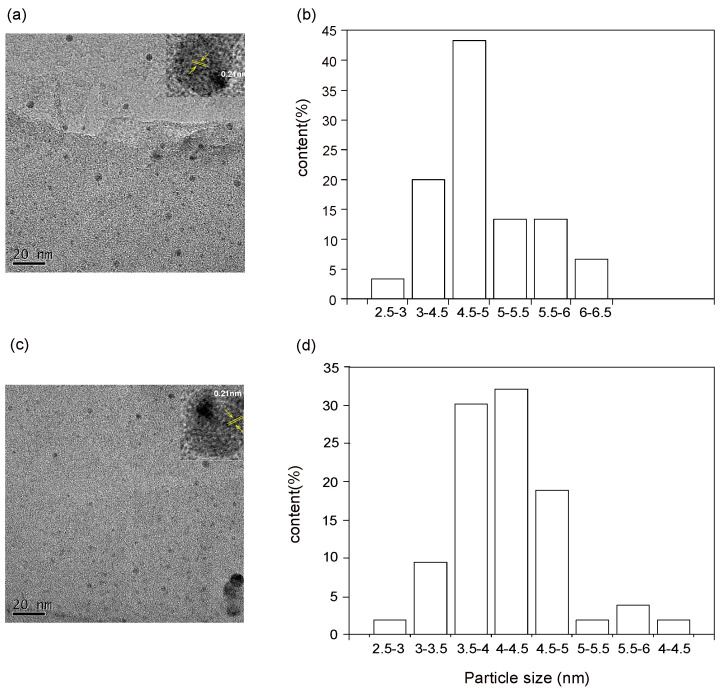
(**a**) TEM image of the JN-CDs, the inset is the high-resolution TEM image of the JN-CDs. Scale bar, 2 nm. (**b**) Size distribution of N-CDs. (**c**) TEM image of the SN-CDs, the inset is the high-resolution TEM image of the SN-CDs. Scale bar, 2 nm. (**d**) Size distribution of N-CDs.

The sizes and morphologies of the N-CDs(JN-CDs and SN-CDs) were observed using TEM [36]. After undergoing solvothermal treatment, nanoscale N-CDs (JN-CDs and SN-CDs) with a uniform structure and shape distribution are observed (Figure 1). The corresponding size distribution results reveal that the average diameter of the prepared JN-CDs is approximately 4.7 nm, while the average diameter of the prepared SN-CDs is around 4.2 nm. The HRTEM analysis of JN-CDs and SN-CDs revealed the presence of a crystalline structure with a lattice spacing of 0.21 nm, resembling the (100) diffraction facets observed in graphite carbon (Figure 1a inset and Figure 1c inset). These synthesized carbon materials partially retain the crystallinity found in raw coal, thereby indicating both short-range order and long-range disorder characteristics to a certain extent. At the nanoscale, they exhibit a remarkable level of structural organization. Elemental analysis data indicate their predominant composition as C, H, and O elements, accompanied by minor quantities of N and S (Table S1).

**Figure 2:**
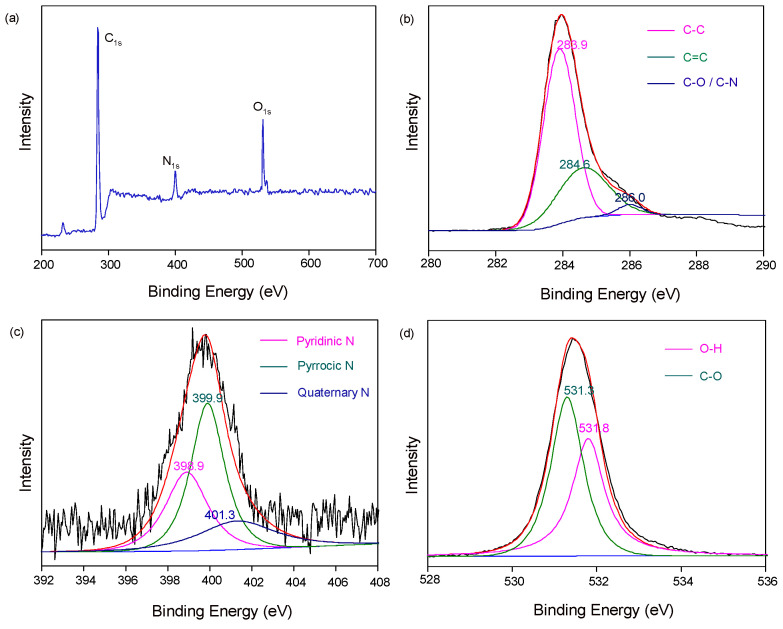
XPS spectra of JN-CDs. (**a**) Survey spectrum; (**b**) C 1s spectrum; (**c**) N 1s spectrum; (**d**) O 1s spectrum.

X-ray photoelectron spectroscopy (XPS) determines the composition and chemical bonding of the sample. The composition and chemical state of elements in JN-CDs and SN-CDs were investigated through XPS analysis [24]. As depicted in Figure 2, JN-CDs exhibit three distinctive peaks at approximately 284.6 eV, 400.0 eV, and 531.0 eV, corresponding to C 1s, N 1s, and O 1s signals, respectively [37]. 

The nitrogen content in N-CDs primarily originates from the precursors of coal and DMF. Notably, JN-CDs display discernible C 1s peaks (Figure 2b) centered around energies of 283.9 eV, 284.6 eV, and 286 eV, respectively, representing C=C, C-C and either C-O or C-N bonds. The N 1s (Figure 2c) peaks are observed at binding energies of 398.7 eV, 399.9 eV, and 401.6 eV, which can be attributed to pyridinic N, pyrrolic N, and quaternary N species, respectively [25]. Furthermore, the O ls peak is deconvoluted into two components: one assigned to the presence of carbonyl groups (C=O) at a binding energy of 531.3 eV and another associated with hydroxyl group (H-O) bonds at a binding energy of 531.8 eV [26].

**Figure 3:**
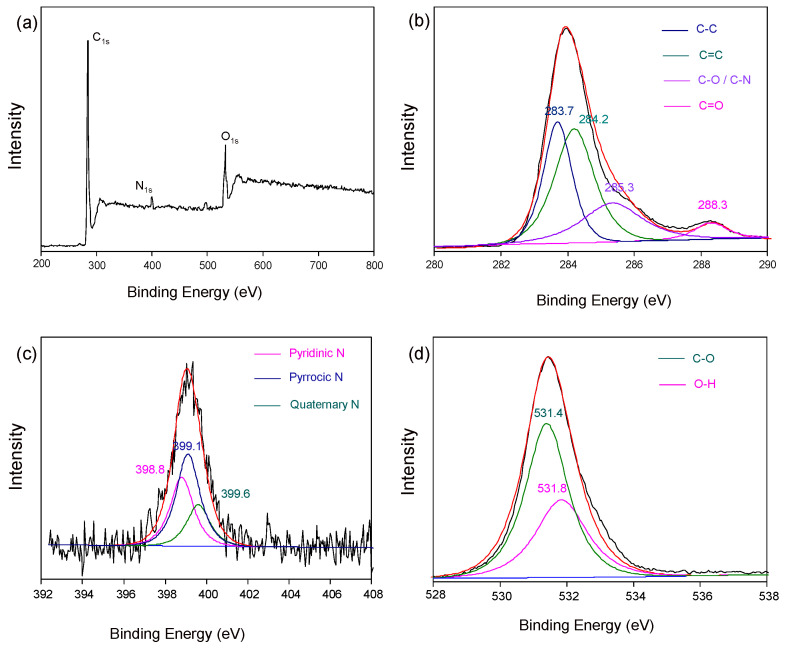
XPS spectra of SN-CDs. (**a**) Survey spectrum; (**b**) C 1s spectrum; (**c**) N 1s spectrum; (**d**) O 1s spectrum.

The high-resolution XPS spectra of SN-CDs (Figure 3) and JN-CDs (Figure 2) exhibit similarities. SN-CDs display distinct C 1s peaks (Figure 3b) at approximately 283.9 eV, 284.6 eV, and 286 eV, corresponding to C=C, C-C and either C-O or C-N bonds, respectively. The N 1s peaks (Figure 3c), centered around 398.7 eV, 399.9 eV, and 401.6 eV, respectively, can be attributed to pyridinic N, pyrrolic N, and quaternary N species. The O 1s peaks can be deconvoluted into C=O bonds at a binding energy of approximately 531.3 eV and C-OH bonds at a binding energy of approximately 531.8 eV [28].

The chemical compositions of JN-CDs and SN-CDs were further determined through elemental analysis. The weight percentages of carbon (C), hydrogen (H), and nitrogen (N) in JN-CDs are approximately 82.83%, 4.48%, and 2.53%, respectively, while the weight percentages of carbon (C), hydrogen (H), and nitrogen (N) in SN-CDs are also about 60.81%, 5.97%, and 2.36%, respectively, as presented in Table S3.

**Figure 4:**
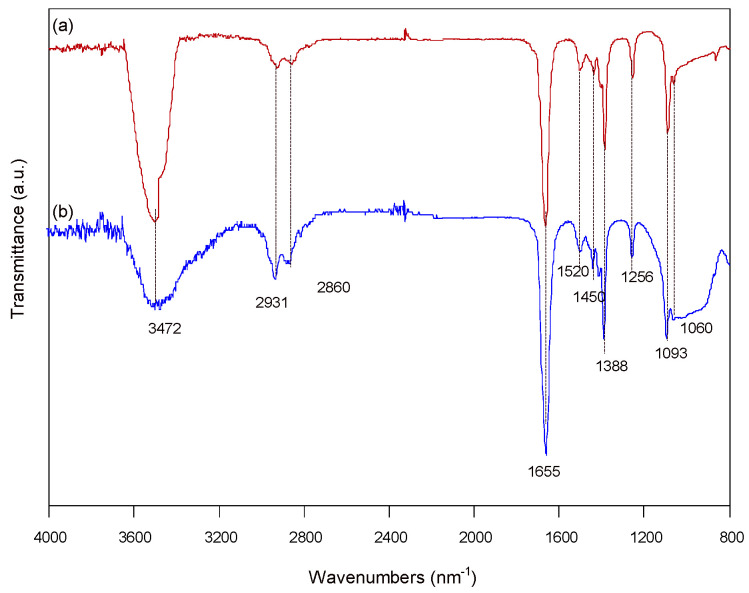
FTIR spectrum of the (**a**) JN-CDs and (**b**) SN-CDs.

The JN-CDs and SN-CDs were subjected to FTIR spectroscopy for characterization, while the chemical composition and surface state of the synthesized carbon dots were meticulously analyzed, as depicted in Figure 4. The figure exhibits distinct vibration patterns corresponding to C=C, C-C, C=O, C-O, and O-H functional groups, indicating the presence of carboxylic acids and other oxygen-containing groups in the carbon sites. Specifically, the prominent peak at a high wavenumber of 3472 cm^−1^ [29] can be attributed to the stretching vibration of O-H bonds. Vibrations observed in the range of 2800 cm^−1^ to 3000 cm^−1^ (2680 cm^−1^, 2931 cm^−1^) correspond to long-chain alkane molecules [30]. The characteristic peaks at 1655cm^−1^ represent stretching vibrations associated with carbonyl groups. The vibration at 1520 cm^−1^ is assigned to respiratory vibrations of C=C bonds within benzene rings. Additionally, vibrations at 1450 cm^−1^ and 1388 cm^−1^ are ascribed to respiratory vibrations of C=C bonds. Stretching vibrations related to C-N bonds occur at 1256 cm^−1^. Furthermore, stretching vibrations corresponding to C-O-C bonds are observed at 1093 cm^−1^ and 1060 cm^−1^. 

In addition, the intrinsic structure of the JN-CDs and single SN-CDs was characterized using Raman spectroscopy (Figure S3). The peaking of the G band around 1570 cm^−1^ indicates the presence of sp2 carbon networks, while the peaking of the D band around 1400 cm^−1^ reflects defects within the graphitized structure. The ratio between the intensity of D peak and G peak (I_D_/I_G_) served as an indicator for assessing the amorphous nature of carbon materials, with a higher I_D_/I_G_ ratio suggesting a greater degree of amorphousness.

**Figure 5:**
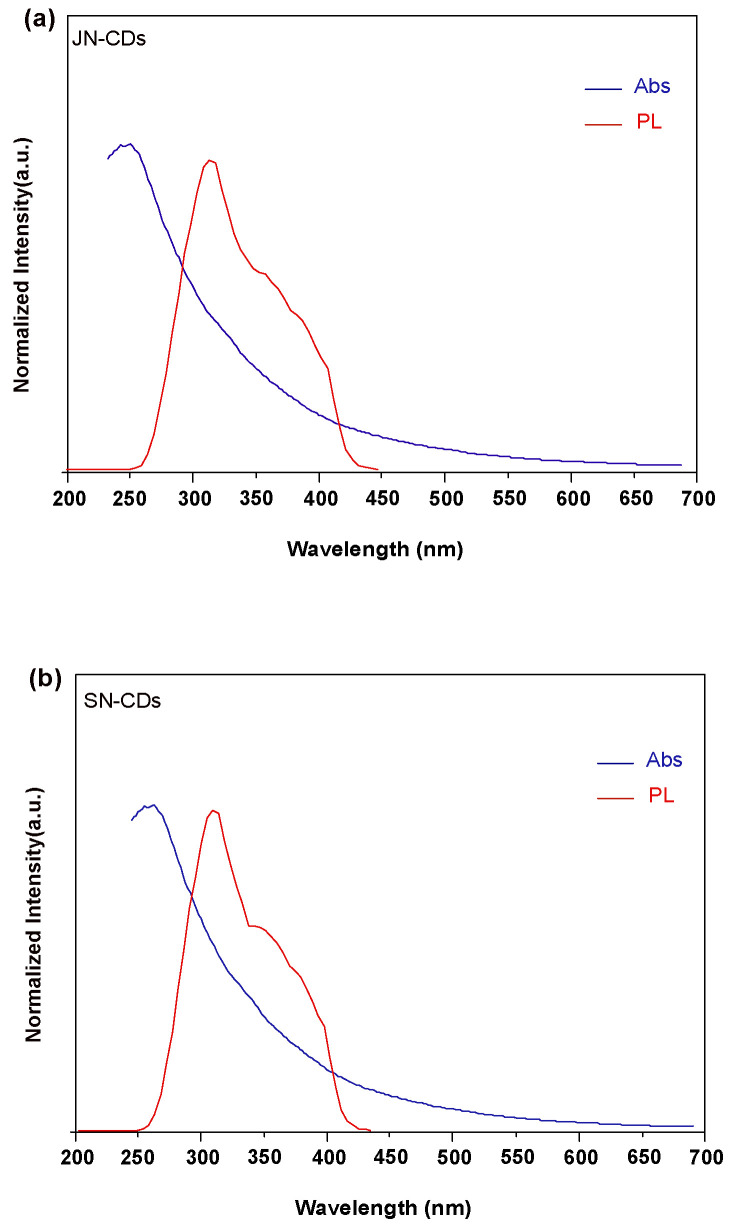
UV-vis absorption and fluorescence emission spectra of (**a**) JN-CDs and (**b**) SN-CDs.

**Figure 6:**
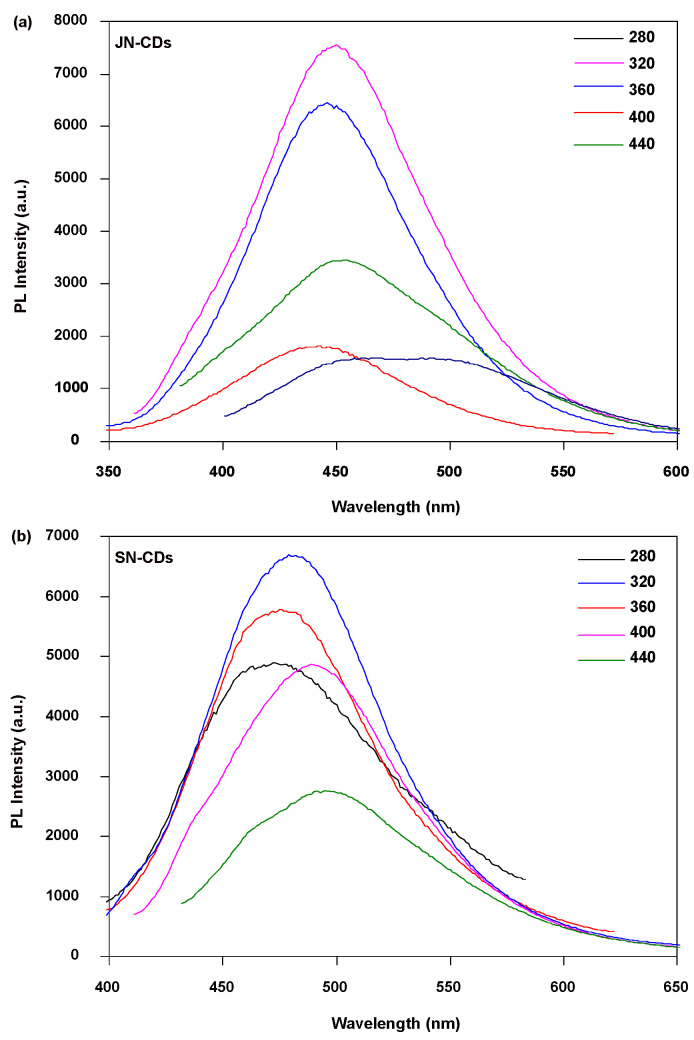
Fluorescence emission spectra of (**a**) JN-CDs and (**b**) SN-CDs at different excitation wavelengths.

The absorption characteristics of JN-CDs and SN-CDs in the UV-vis spectrum are clearly evident in Figure 5, becoming more pronounced as the wavelength decreases. A prominent peak near 270 nm can be attributed to the π-π* interaction of aromatic compounds and the n-π* transition of oxygen-containing functional groups. However, there is relatively weak absorption in visible light without any distinct peaks observed. The PL excitation spectrum depicted in Figure 5 exhibits a well-defined excitation peak at 310 nm, corresponding to the UV-vis spectrum. Additionally, a slightly weaker shoulder appears at a wavelength of 370 nm. Notably, SN-CDs exhibit a slightly stronger shoulder at 370 nm compared to JN-CDs. When excited at a wavelength of 310 nm, JN-CDs emit an emission spectrum peak of around 450 nm, while SN-CDs emit around 475 nm (Figure 6). The solvothermal method employed for synthesizing coal-based carbon dots is essentially a wet chemical preparation technology; hence, resulting carbon dots are dispersed within the solvent system and form a stable sol system in DMF. Upon dilution under visible light, the solution containing prepared coal-based carbon dots appears brown-yellow in coloration. Under excitation with ultraviolet light at 360 nm, these fluorescent carbon dots emit bright blue fluorescence similar to most fluorescent carbon dots reported in the literature [32].

As Figure 6 shows, both JN-CDs and SN-CDs are excited at different wavelengths from 280 to 440 nm. For JN-CDs, emission bands vary according to the excitation wavelength, similar to the PL spectra of SN-CDs [33].

The JN-CDs and SN-CDs synthesized in this study exhibit excitation-dependent photoluminescence behavior (Figure 6), consistent with previously reported observations on the majority of photoluminescent carbon dots (PL CDs). 

The luminescent properties of carbon dots were investigated at various excitation wavelengths, and the fluorescence behavior of pristine carbon dots was found to be reliant on the excitation light source. With an increase in excitation wavelength, a rapid decline in intensity is observed, accompanied by a gradual redshift of the fluorescence peak position. This trend resembles that observed for coal-based carbon dots prepared via the acid oxidation method discussed earlier. Notably, when excited at a 320 nm wavelength, these carbon dots exhibit their highest fluorescence intensity, which aligns with the findings in Figure 5 show casing their fluorescence excitation and emission spectra.

The PL emission and excitation spectra are presented in Figure 6. Both JN-CDs and SN-CDs exhibit fluorescence behavior that is dependent on the excitation light. Both JN-CDs and SN-CDs display a blue color, characterized by broad peaks and narrow peaks. Furthermore, the PL emission peaks of JN-CDs and SN-CDs are observed at 450 nm and 475 nm, respectively. All N-CDs’ PL emission spectra demonstrate a slight shift with varying excitation wavelengths, indicating uniformity in the size and surface state of sp2 domains within N-CDs. The intensity decreases rapidly as the excitation wavelength increases, while the fluorescence peak gradually redshifts with increasing excitation wavelengths, resembling coal-based carbon dots prepared via the acid oxidation method. This redshift may be attributed to an enlargement of the sp2 domain or binding of effective conjugation length to surface groups. It can be inferred that tunable PL emission can be achieved through control over sp2 sites, which relies on the size, shape, and fraction of sp2 domains. Interestingly, both JN-CDs and SN-CDs emit light under short-wavelength excitations (SWLEs) due to specific fine structures, suggesting a similar fluorescence emission mechanism. Notably, the enhanced fluorescence intensity of JN-CDs primarily arises from their more conjugated structure compared to SN-CDs.

To further investigate the superiority of the solvothermal method, coal-based carbon dots (CDs) were prepared via chemical oxidation in hydrogen peroxide. Although single coal-based CDs exhibit a smaller size distribution than those obtained by solvothermal treatment (Figure S2), their quantum yield (QY) is relatively low at 0.81% [2]. This can be attributed to the abundant oxygen-containing functional groups and less conjugated structure resulting from strong oxidation, which promotes the nonradiative recombination of electron-hole pairs and thus leads to a lower QY.

**Figure 7:**
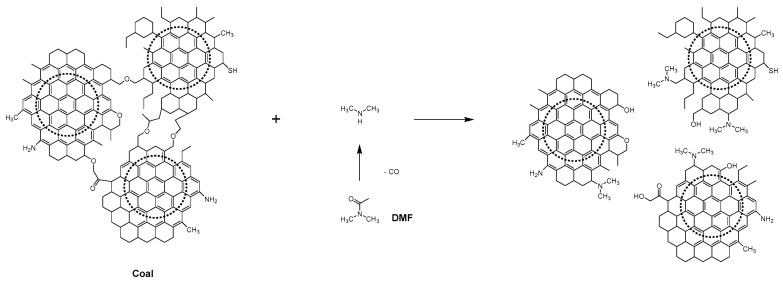
Schematic illustration of a possible mechanism for forming N-CDs.

The results further demonstrate that coal serves as an ideal and distinctive carbon source for synthesizing CDs with high quantum yield (QY). It is well known that anthracite possesses a macromolecular structure primarily composed of sp2 carbon domains and adjacent layers connected by bridging bonds (Figure 7). During the solvothermal process, the light components in anthracite are effectively dispersed in the solvent.

Meanwhile, DMF can be partially decomposed into dimethylamine under the condition of a microwave, and dimethylamine can be bonded to the sp2 carbon domain through the nucleophilic ring-opening reaction with the bridge bond between the adjacent layers. DMF can undergo partial decomposition to yield dimethylamine, which can be incorporated into the sp2 carbon domains through nucleophilic ring-opening reactions with the bridging bonds between adjacent layers. This process facilitates the exfoliation of small-sized sp2 carbon structures from anthracite and promotes the formation of coal-based N-CDs [34]. In comparison to CDs synthesized via oxidation treatment, solvothermal synthesis yields N-CDs with relatively fewer oxygen-containing functional groups and a more pronounced conjugated structure.

Therefore, the dominance of nonradiative recombination in electron-hole pairs is not significant, thereby resulting in a high quantum yield (QY). Additionally, the energetically favorable subdomains formed through solvothermal treatment contribute to the enhanced QY and, consequently, exhibit strong intrinsic emission. 

The quantum yield (QY) of coal-based nitrogen-doped carbon dots (JN-CDs) and sulfur-doped carbon dots (SN-CDs), obtained through microwave solvothermal synthesis, reaches an impressive 49.5% and 40.0%, respectively, surpassing that of CDs synthesized via oxidation and surface modification techniques (Table S4).

The yield and quantum yield of coal-based carbon points appear to be inversely correlated, with high values of one parameter corresponding to low values of the other. The presence of amorphous carbon in the product may play a significant role in this relationship. The characterization of coal-based carbon points in the provided figure reveals that the obtained products generally contain a certain amount of amorphous carbon. Low metamorphic subbituminous coal promotes higher product yield, while high metamorphic anthracite enhances quantum yield. This can be attributed to the underdeveloped hexagonal network structure and larger proportion of amorphous carbon in low-metamorphic bituminous coal, which allows for its retention during production under moderate oxidizing conditions, resulting in increased yield but lower quantum efficiency due to a lack of photoluminescence properties [2,5].

**Figure 8:**
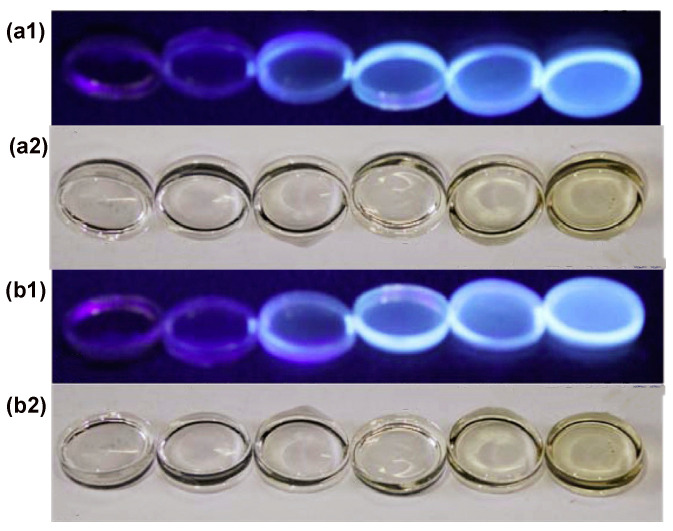
Digital photographs of the JN-CDs-PMMA and SN-CDs-PMMA composites captured under (**a1**,**b1**) 365 nm excitation and (**a2**,**b2**) visible light.

The coal-based JN-CDs and SN-CDs are uniformly dispersed within the PMMA matrix, resulting in a cohesive structure that effectively preserves the inherent advantages of their respective matrices (Figure 8). The concentrations of JN-CDs and SN-CDs were 0, 2.5, 25, 50, 100, and 200 ppm from left to right in the experimental setup. 

In this experiment, coal-based nitrogen-doped carbon dots (JN-CDs and SN-CDs) were dispersed in polymethyl methacrylate (PMMA) at concentrations ranging from 0 to 200 ppm, as depicted in the digital photo in Figure 8. As illustrated in the figure, an increase in N-CD (JN-CDs and SN-CDs) concentration up to 50 ppm resulted in a light brown coloration of the plexiglass under visible light, while further increments up to 200 ppm deepened the color visibly. Under UV irradiation, it was observed that pristine PMMA exhibited no fluorescence, whereas increasing N-CD (JN-CDs and SN-CDs) concentrations led to a gradual enhancement of fluorescence until reaching a maximum of 200 ppm without any noticeable quenching phenomenon. However, no significant visual differences were observed when comparing JN-CDs-PMMA and SN-CDs-PMMA [1].

## 3. Materials and Methods

### 3.1. Materials

Two types of Chinese coal were utilized in this study. Shaerhu lignite was sourced from Shaerhu Coal Industry, Xinjiang, China, while Jin 15 Anthracite was obtained from Jincheng Coal Industry, Jincheng, China. The proximate and ultimate analyses of these coal samples are presented in Table S1. Prior to experimentation, the coal samples were crushed and sieved to a particle size of 200 mesh (74 μm) and subsequently dried. All chemicals employed in this study were of ultrapure analytical grade and procured from Shanghai Damao Chemical Reagent Factory, Shanghai, China. No further purification was performed on any reagents used. Double distilled water was utilized throughout all experiments [2].

### 3.2. Synthesis of the of JN-CDs and SN-CDs 

Shaerhu lignite and Jin 15 Anthracite were crushed and dispersed in 100 mL of DMF at a concentration of 2 mg/mL, respectively. Following ultrasonic crushing for 0.5 h, the mixture was transferred to a microwave reactor maintained at a power level of 800 W for 6 h. After cooling down, centrifugation was performed to obtain a brown colloid supernatant. CD powder was obtained by rotary evaporation of the solution, referred to as SN-CDs and JN-CDs. In order to establish a control group, a comparative experiment without coal was conducted [3,14].

### 3.3. Synthesis of N-CDs–Polymethyl Methacrylate (N-CDs-PMMA) Composites

A small quantity of AIBN was dispersed into methyl methacrylate (5 mL) as an initiator using ultrasonic treatment. Subsequently, the N-CDs synthesized in Section 3.2 were added to synthesize the N-CDs-PMMA composite monoliths by solidifying at 60 °C for 24 h. The loading fractions of N-CDs in PMMA were precisely controlled at levels of 0, 2.5, 25, 50, 100, and 200 ppm, respectively. These composites were designated as SN-CDs-PMMA and JN-CDs-PMMA [1].

### 3.4. Characterization

The N-CDs were synthesized using a microwave reactor (XH-300C, Beijing Xianghu, Beijing, China), and the morphology of coal was characterized by scanning electron microscopy (SEM, Quanta 250, FEI, Hillsboro, OR, USA). The structure of N-CDs was examined using transmission electron microscopy (TEM, Tecnai G2F20, FEI, Hillsboro, OR, USA) and high-resolution transmission electron microscopy (HRTEM, Tecnai G2F30S-Twin, FEI, Hillsboro, OR, USA). Fourier-transform infrared (FTIR) spectra were obtained using Nicolet IR-560, (Thermo Scientific, Shanghai, China). UV-vis absorption and fluorescence spectra were recorded on a Thermofisher UV-vis spectrophotometer and a Thermofisher spectrophotometer (Thermo Scientific, Shanghai, China), respectively. Data from X-ray photoelectron spectroscopy (XPS) were collected using an X-ray photoelectron spectrometer (Thermofisher ESCALAB 250Xi, Thermo Scientific, Shanghai, China), while Raman measurements were carried out on Raman Spectroscopy Senterra (Bruker, Germany) [27,30].

### 3.5. Quantum Yield Measurements

Quantum yield (QY) determination was conducted following a previously established procedure. Quinine sulfate (literature QY 0.54 at 340 nm), anthracene literature (QY 0.27 at 340 nm), and Rhodamine B (literature QY 0.31 at 340 nm) were chosen as reference standards for comparison [10]. The QYs of the coal-based CDs were calculated by comparing their integrated photoluminescence intensity (excited at 340 nm) and absorbance value (at 340 nm) with those of the reference standards using the following equation [1,2,3]:

(1) $$\emptyset =\emptyset_{r}\times \frac{A_{r}}{I_{r}}\times \frac{I}{A}\times \frac{n^{2}}{n_{r}^{2}}$$

This equation represents the quantum yield, and ‘I’ denotes the measured integrated emission intensity, ‘n’ stands for refractive index, and ‘A’ represents optical density. The subscript ‘r’ refers to the known quantum yield of the reference fluorophore. To minimize re-absorption effects, absorbance in the fluorescence cuvette with a path length of 10 mm was maintained below 0.1 at an excitation wavelength of 340 nm.

## 4. Conclusions

In summary, we have developed a facile process for synthesizing fluorescent nitrogen-doped carbon dots (N-CDs) from coal (Jin 15 Anthracite and Shaerhu lignite) as raw materials and dimethyl formamide (DMF) as the solvent using a microwave pyrolysis method. The resulting JN-CDs and SN-CDs exhibit strong photoluminescence properties, which can be attributed to the facile exfoliation of smaller sp2 carbon structures from the coal during the synthesis process. The decomposition of DMF under microwave conditions leads to the formation of dimethylamine, which can subsequently undergo a nucleophilic ring-opening reaction with the bridge bond between adjacent layers, enabling its bonding to the sp2 carbon domain [38]. Our results demonstrate that these JN-CDs and SN-CDs hold great potential for bioimaging applications, with JN-CDs exhibiting a high quantum yield (QY) of 49.5% and a production yield of 25.6%, while SN-CDs show a QY of 40% and a production yield of 35.1%. It is worth noting that the production yield and quantum yield are inversely related in coal-based carbon dots; thus, subbituminous coal with a lower metamorphic degree favors higher product yields, whereas anthracite with a higher metamorphic degree enhances quantum yields [39]. Therefore, we propose and discuss a possible mechanism for N-CD formation.

## Figures and Tables

**Table 1:** Photophysical parameters of the JN-CDs, SN-CDs, and carbogenic dots measured in DMF.

	λ_abs_ nm ^a^	λ_ex_ nm ^b^	λ_em_ nm ^c^	Φ (%)	Yield
JN-CDs	270	310	445	49.5	25.7
SN-CDs	270	310	465	40.0	35.1
Carbogenic dots	275	365	445	18.6	-

^a^ Absorption maxima. ^b^ Excitation wavelength maxima. ^c^ Emission wavelength maxima.

## Data Availability

Data are contained within the article and Supplementary Materials.

## Supplementary Materials

The following supporting information can be downloaded at https://www.mdpi.com/article/10.3390/molecules29061349/s1, Figure S1: SEM image of (a) Jin 15 Anthracite and (b) Shaerhu lignite; Figure S2: (a) TEM image of the r-CDs, the inset is the high-resolution TEM image of the r-CDs. Scale bar, 2 nm. (b) Size distribution of r-CDs. Figure S3: Raman spectra of JN-CDs and single SN-CDs. Table S1: Proximate and ultimate analyses (wt%) of samples; Table S2: Quantum yield of the coal-based CDs; Table S3: Elemental analysis data (wt%) of samples; Table S4: Comparison of QY for some coal-based CDs.
