# Supplementary material for: Microwave-Assisted Grafting of Coal onto Nitrogen-Doped Carbon Dots with a High Quantum Yield and Enhanced Photoluminescence Properties
Source: Molecules. 2024 Mar 18;29(6):1349. doi: 10.3390/molecules29061349 (PMC10974261; doi:10.3390/molecules29061349)
Supplement: Supplementary file 1 [file molecules-29-01349-s001.zip › molecules-2919792-supplementary.pdf]

Supplementary Materials

Microwave-Assisted Grafting of Coal onto Nitrogen-Doped Carbon Dots with a High Quantum Yield and Enhanced Photoluminescence Properties

Chong Shi [1] and Xian-Yong Wei [1,2,3,*]

[1] Jiangsu Province Engineering Research Center of Fine Utilization of Carbon Resources and Key Laboratory of Coal Processing and Efficient Utilization, Ministry of Education, China University of Mining & Technology, Xuzhou 221116, Jiangsu, China; shichong0901@163.com (C.S.)

[2] Key Laboratory of Chemistry and Chemical Engineering on Heavy-Carbon Resources, Yili Normal University, Yining 835000, Xinjiang, China

[3] State Key Laboratory of Chemistry and Utilization of Carbon-Based Energy Resources Jointly Built by Xinjiang Uyghur Autonomous Region and Ministry of Science and Technology, Key Laboratory of Coal Clean Conversion & Chemical Engineering Process (Xinjiang Uyghur Autonomous Region), College of Chemical Engineering, Xinjiang University, Urumqi 830046, Xinjiang, China

* Correspondence: wei_xianyong@163.com (X. Y. Wei); Tel: +86 516 83885951; fax: +86 516 83884399

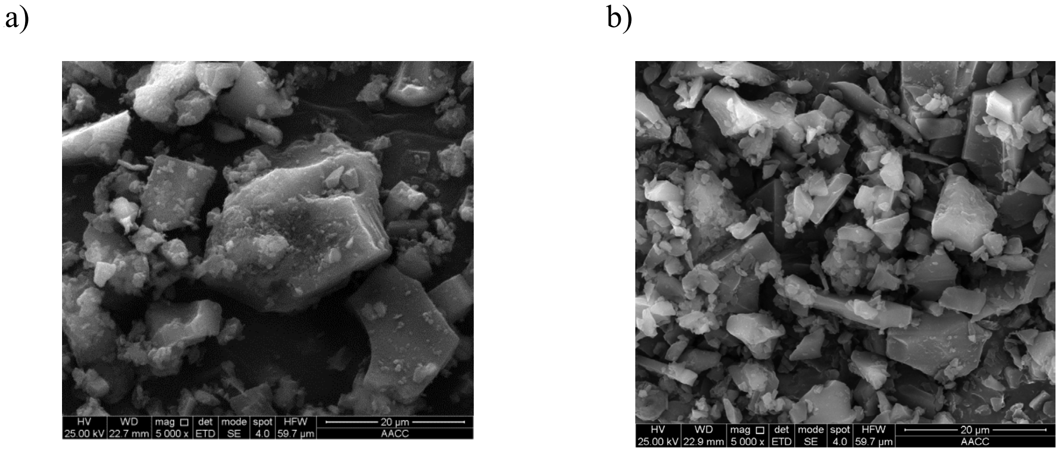

Figure S1. SEM image of a) Jin 15 Anthracite and b) Shaerhu lignite.

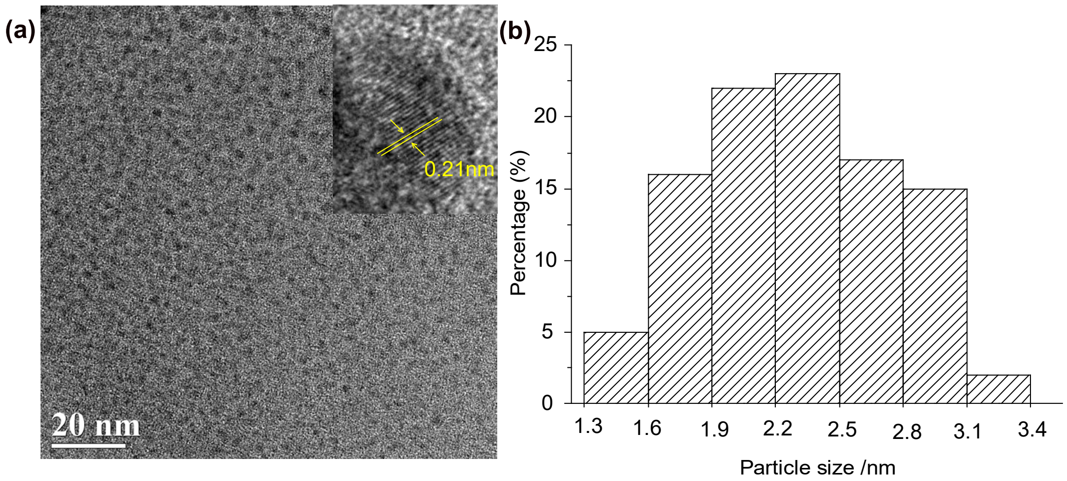

Figure S2. (a) TEM image of the r-CDs, the inset is the high-resolution TEM image of the r-CDs. Scale bar, 2 nm. (b) Size distribution of r-CDs.

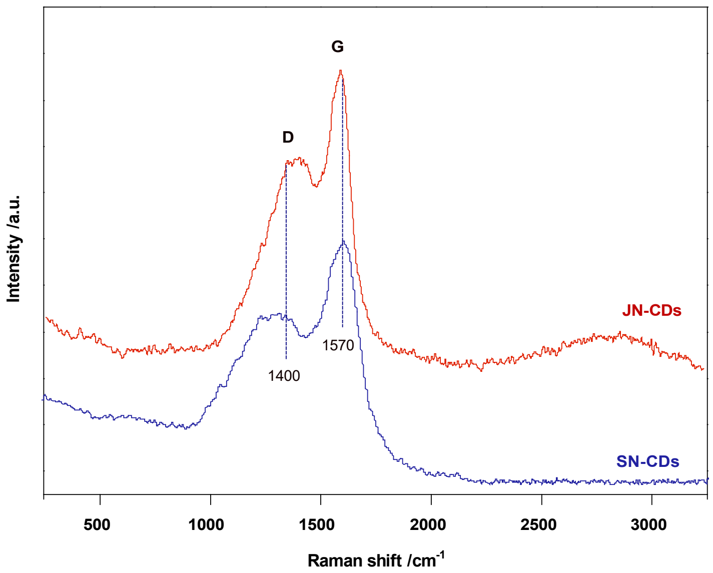

Figure S3. Raman spectra of JN-CDs and single SN-CDs.

Table S1. Proximate and ultimate analyses (wt%) of samples.

Sample name	Proximate analysis			Ultimate analysis (daf)			
	M_{ad}	A_{d}	V_{daf}	C	H	N	O_{diff}
Jin 15 Anthracite	2.31	39.73	6.86	88.40	1.95	0.92	>4.54
Shaerhu lignite	12.20	5.31	37.91	66.81	3.37	0.66	>29.09

Table S2. Quantum yield of the coal-based CDs.

Sample	Integrated emission intensity (I)	Abs. at 340 nm (A)	Refractive index of	Quantum yield	Yield (wt.%)	Reference
Rhodamine B	253593	0.0714980	1.33	31	-	
JN-CDs	337947	0.59668	1.33	49.5	25.7	
SN-CDs	2778079	0.60702	1.33	40	35.1	
Coal-based N-CDs	323836.9	0.059702	1.33	47	25.6	[1]
Carbogenic dots	149443	0.069867	1.33	-	18.6	[1]
Single coal-based dots/Rhodamine B	50088.17/3708133.79	0.066697/0.054217	1.33	-	0.81	[2]

Table S3. Elemental analysis data (wt%) of samples.

Sample name	Ultimate analysis (daf)				
	**C**	**H**	**N**	**S**	**O_{diff}**
JN-CDs	82.83	4.48	2.53	1.08	>4.54
SN-CDs	60.81	5.97	2.36	0.96	>29.09

Table S4. Comparison of QY for some coal-based CDs.

**Sample**	**Integrated emission intensity (*I*)**	**Abs. at 340 nm (*A*)**	**Refractive index (*n*)**	**Quantum yield (*Φ*, %)**	**Reference**
JN-CDs	337947	0.59668	1.33	49.5	
SN-CDs	2778079	0.60702	1.33	40.0	
N-CDs	251593.1	0.070387	1.33	47.0	[1]
r-CDs	44233	0.055247	1.33	8.1	[2]
QDs (NaOH treatment)	—	—	1.33	8.1	[3]
EDA-CDs	5333 (at 340 nm)	0.04905	1.33	18.6	[4]

References

1. Li, M.Y.; Yu, C.; Hu, C. Solvothermal conversion of coal into nitrogen-doped carbon dots with singlet oxygen generation and high quantum yield. *Chem. Eng. J.* **2017**, *320*, 570–575.
2. Shi, C.; Wei, X.Y.; Liu, F.J. Carbon Dots Derived from Facile Tailoring of Shaerhu Lignite as a Novel Fluorescence Sensor with High-Selectivity and Sensitivity for Cu^{2+} Detection *ChemistrySelect* **2020**, *5*, 12125–12130.
3. Srivastava, A.K.; Dev, A.; Karmakar, S. Nanosensors and nanobiosensors in food and agriculture. *Environ. Chem. Lett.* **2017**, *16*, 161–182.
4. Zhou, T.; Zhang, J.; Liu, B.; Wu, S.; Liu, J. Nucleoside-based fluorescent carbon dots for discrimination of metal ions. *J. Mater. Chem. B* **2020**, *8*, 3640–3646.
